# Corporate Competing Culture and Environmental Investment

**DOI:** 10.3389/fpsyg.2021.774173

**Published:** 2022-01-20

**Authors:** Jinfang Tian, Wei Cao, Qian Cheng, Yikun Huang, Shiyang Hu

**Affiliations:** ^1^School of Statistics, Shandong University of Finance and Economics, Jinan, China; ^2^School of Public Affairs Administration, China Agriculture University, Beijing, China; ^3^School of Economics and Business Administration, Chongqing University, Chongqing, China

**Keywords:** competing culture, environmental investment, internal control quality, corporate sustainable development, MD&A

## Abstract

Using Chinese listed companies as research setting, this paper constructs a measure of corporate competing culture through textual analysis on firms’ management discussion and analysis (MD&A) disclosures, and examines the impact of corporate competing culture on environmental investment. The results show that competing culture has a significant and positive impact on firms’ environmental investment, and the results remain robust to a battery of robustness tests. Moreover, the mediating analysis indicates that competing culture promotes corporate environmental investment through enhancing firms’ internal control quality. Furthermore, the heterogeneity results show that the positive impact of corporate competing culture on environmental investment is more pronounced in firms with larger size, stronger corporate governance, in high-polluting industry, and located in less developed regions. Our findings shed light on the importance of corporate competing culture and provide practical implications for corporate sustainable development.

## Introduction

Corporate culture is *a set of norms and values that are widely shared and strongly held within an organization* (Guiso et al., 2015). Positive corporate culture can foster a healthy working environment by enhancing internal communication (Jacobs et al., 2013), which in turn shapes employee mindsets, enhances corporate strategic decision-making, and ultimately increases firm value (Yusuf, 2002). As such, a sound corporate culture is conducive to corporate management and operation (Han, 2004).

Competing culture is an important type of corporate culture (Fiordelisi and Ricci, 2014). It refers to a culture that upholds competing components and seeks to make the company more competitive as a whole (Fiordelisi et al., 2019). Employees may feel a sense of pressure in a competing culture, which may increase internal competition and, as a result, has an influence on the company’s market share and profitability (Fiordelisi et al., 2019). Competing culture, if properly guided by managers, will motivate employees’ productivity and enhance firms’ core competencies (Fiordelisi et al., 2019); otherwise, it may cause cut-throat internal competition and conflicts, resulting in a loss in firms’ core competencies (Hu et al., 2021).

Corporate environmental investment plays a non-negligible role in promoting sustainable development (Tian et al., 2020). Companies can improve environmental performance and reduce environmental liabilities by investing in environmentally friendly technologies that reduce emissions and improve resource utilization (Bierbaum et al., 2019). Corporate environmental investment is a major part of corporate social responsibility (Bierbaum et al., 2019). By investing in pro-environmental activities, companies can improve their social reputation (Aksak et al., 2016), gain the trust of stakeholders, and therefore enhance their financial performance (Pekovic et al., 2018). As such, corporate environmental investment is conducive to corporate sustainable development (Tian et al., 2020).

A number of factors, including external and internal factors, can influence corporate environmental investment. External factors include environmental regulations (Huang and Lei, 2021), government subsidies (Jung and Feng, 2020), and market competition (Ducassy and Montandrau, 2015). First, in terms of environmental regulations, considering that companies are the main carbon emitters and energy consumers (Alam et al., 2019), governments of various countries have introduced environmental laws and regulations to regulate companies’ operation and production (Du et al., 2020), forcing companies to improve their environmental performance. Second, in terms of government subsidies, the governments may provide incentives, such as green subsidies, for companies to adopt environmentally friendly strategies (Huang et al., 2020), leading to an increase in corporate environmental investment. Third, in terms of market competition, companies in the same industry can also incentivize peer companies’ environmental investment by investing in clean technologies to increase core competencies and gain competitive advantages in the market (Sengupta, 2015). Internal factors include board structure (Du et al., 2017; Sun et al., 2020), manager characteristics (Wei and Zhou, 2020), and corporate culture (Fiordelisi et al., 2019). First, in terms of board structure, the more diverse the board members’ background and educational attainment, the more feasible the environmental investment decisions made by the firm (Du et al., 2017; Sun et al., 2020). Second, in terms of manager characteristics, managers’ insight and personality have a substantial impact on firms’ decisions of long-term investment such as environmental investment (Wei and Zhou, 2020). Third, in terms of corporate culture, Fiordelisi et al. (2019) find that corporate culture plays a guiding role in the strategic decision making of environmental investment.

Albeit rarely studied, corporate culture, as an internal factor, could play an important role in firms’ strategic decision-making process of environmental investment (Lu and Wang, 2021). Thus, corporate competing culture, as an important corporate culture, might have a significant relation with corporate environmental investment that merits investigation. We select Chinese firms as the research setting to examine impact of competing culture on corporate environmental investment because China is the largest carbon emitter and listed firms contribute most to a nation’s carbon emissions (Chen et al., 2021; Fan et al., 2021; Frank et al., 2021; Yan et al., 2021). Accordingly, we select Chinese listed firms from 2010–2019 to investigate the relationship between corporate competing culture and environmental investment. Based on the management discussion and analysis (MD&A) disclosures of Chinese listed companies, this paper uses text mining technique to construct a quantitative measure of corporate competing culture and examines the impact of competing culture on corporate environmental investment. The results show that corporate competing culture has a significant and positive impact on environmental investment. This positive impact is more pronounced in companies with larger size, stronger corporate governance, in high-polluting industry, and located in less developed regions. Through the mediating analysis, we find that internal control quality transmits the positive impact of corporate competing culture onto environmental investment. The results remain robust to multiple robustness tests. Therefore, this study provides practical implications for promoting sustainable development of businesses and the society.

The remainder of the paper is structured as follows. Section “Literature Review” reviews related literature on corporate competing culture and environmental investment, followed by data and variable descriptions in section “Research Design.” Section “Results” shows the results and section “Conclusion” concludes the paper.

## Literature Review

### Corporate Competing Culture

Corporate culture is *a set of norms and values that are widely shared and strongly held within an organization* (Guiso et al., 2015). Through shared values and norms, corporate culture can enhance the effectiveness of internal communication (Jacobs et al., 2013) and motivate employees to work toward common goals (Deal and Kennedy, 1983). More importantly, corporate culture influences the value of a company by influencing employees’ mindsets and work productivity (Fiordelisi et al., 2019). Typical corporate cultures include innovation culture (Fiordelisi et al., 2019), integrity culture (Peng et al., 2020), and many more.

A culture of innovation inspires and promotes creative thinking and action among members of an organization, allowing them to produce remarkable results (Michaelis et al., 2018). A cautionary example is the company *Kodak*, where rigid bureaucracy and fixed mindsets of top management, and lack of creative thinking have greatly hampered the company’s development of image capturing and sharing technologies. *Kodak*’s inability to adapt to innovative digital thinking significantly reduced its market share, stock prices, and market value, eventually resulting in its bankruptcy (Lucas and Goh, 2009).

An integrity-oriented culture encourages companies to take social responsibility and gain public trust so as to increase their social and economic value (Peng et al., 2020). The energy company *Enron*, however, abandoned the culture of integrity and resorted to deceive the investors by manipulating financial reports, resulting in a plunge in its stock price and, and eventually, a bankruptcy (Linthicum et al., 2010).

Few existing studies investigate corporate competing culture. A competing culture is a culture that incorporates high social comparison (Hofstede, 1986). Social comparison refers to individuals comparing their own beliefs, attitudes and achievements with those of others (Buunk and Gibbons, 2007). Such social comparisons occur between individuals, and between companies (Hofstede, 1986). When such social comparisons take place within companies and create differences, a competing “motivation field” is created (Dissanaike et al., 2019), resulting in a competing culture. Corporate competing culture refers to a consensus and atmosphere within a firm that upholds competition components in order to make the firm competitive as a whole (Fiordelisi et al., 2019). Companies pursuing a competing culture are often distinguished by a focus on competitiveness enhancement, customer centricity, and effective internal and external controls (Fiordelisi et al., 2019). Moreover, competing culture increases mutual supervision and competition among employees and teams, creating a sense of urgency and pressure (Fiordelisi et al., 2019). If properly guided by the managers, it can motivate employees to create and work actively (Huarng and Mas-Tur, 2015), improve the quality and efficiency of production, and make the firm more competitive in the market (Fiordelisi et al., 2019). However, if the managers have a poor guidance of competing culture, for example, by prioritizing short-term interests over long-term growth, it can result in cut-throat competition, low trust, high interpersonal sensitivity within the firm. This may cause further conflicts, making it difficult for strategic decision making and heightening the company’s operation cost (Hu et al., 2021).

### Corporate Environmental Investment

Corporate social responsibility (CSR) refers to a range of initiatives and practices that firms voluntarily adopt to meet social and environmental requirements and contribute to environmental sustainability (Barbosa and Oliveira, 2020). CSR is the aggregation of the obligations that society as a whole expects companies to fulfill, i.e., the responsibilities that companies have toward other stakeholders in society (Carroll, 1979). CSR encompasses four categories, including economic responsibility, legal responsibility, ethical responsibility, and philanthropic responsibility (Carroll, 1979). By practicing CSR, companies can improve their reputation, gain the trust of stakeholders, generate more social value, and thereby increase firm value (Barbosa and Oliveira, 2020; Su et al., 2020). The most common forms of CSR activities include environmental protection, ethical labor practices, and community services (Barbosa and Oliveira, 2020). Among them, environmental investment is an important way for companies to practice CSR and contribute to environmental protection.

Environmental investment is a type of investment that aims to solve real or potential environmental problems and to balance the relationship between humans and the environment (Linhard, 2005). Typical environmental investment includes expenditure on research and development, renovation of environmental technologies, renovation of environmental facilities, pollution control, ecological protection, and cleaner production (Askildsen et al., 2006; Kumari et al., 2021). Environmental investment helps to balance the human-environment connection and promotes sustainable development (Tian et al., 2020). Corporate environmental investment promotes the adoption of green technologies, which lead to efficient resource use and lower environmental compliance cost (Bierbaum et al., 2019), thereby improving corporate environmental performance (Tian et al., 2020). In addition, companies can fulfill their social responsibility through environmental investment (Bierbaum et al., 2019), which improves firm reputation (Aksak et al., 2016), brand value (Guenther and Guenther, 2019), and overall corporate performance (Pekovic et al., 2018). On the other hand, the main objective of companies is to generate profits and maximizes shareholder interests (Murthy et al., 2021); however, environmental investment—as a form of public utility investment (Michelfelder et al., 2019)—has lower returns and higher costs in the short term (Wei and Zhou, 2020). As such, firms’ budgets may be constrained and production and operation may be affected by environmental investments (Azadegan et al., 2018). Therefore, the motivations of environmental investment from the private sector deserve to be further explored.

We classify driving factors of corporate environmental investment into external and internal factors. External factors include environmental regulations (Huang and Lei, 2021), government subsidies (Jung and Feng, 2020), and market competition (Ducassy and Montandrau, 2015). First, nowadays, global environmental problems are increasingly severe, and firms are the main carbon emitters and energy consumers (Alam et al., 2019). Given that industrial production depends heavily on environmental resources (Yin et al., 2021), governments worldwide have introduced various environmental regulations and policies to regulate industrial production (Du et al., 2020), forcing companies to increase investment in environmental protection. Second, green subsidies can incentivize corporate environmental investment (Jung and Feng, 2020). Green subsidy refers to the government’s provision of loans, tax subsidies, and/or other incentives for firms to adopt environmentally-friendly measures (Huang et al., 2020) and increase environmental investment. Third, when companies make profits from investing in new environmental technologies such as clean technologies, it will create an appealing incentive for other companies in the market to increase environmental investment to compete (Sengupta, 2015).

Internal driving factors of corporate environmental investment include board structure (Du et al., 2017; Sun et al., 2020), manager characteristics (Wei and Zhou, 2020), and corporate culture (Fiordelisi et al., 2019). First, Sun et al. (2020) finds that board member diversity, such as gender and age, increases the diversification of firms’ investment portfolio (Du et al., 2017; Sun et al., 2020) and promotes environmental investment. Second, managers with short-sightedness characteristics tend to prioritize short-term investment for immediate benefits over long-term and sustainable investment such as environmental investment (Wei and Zhou, 2020). Third, corporate culture plays a guiding role in firms’ strategic decision making (Fiordelisi et al., 2019). However, research on corporate culture remains scarce, especially on the influence of corporate competing culture on environmental investment.

### Corporate Competing Culture and Environmental Investment

Existing research shows mixed results on the impact of competing culture on corporate environmental investment. On the one hand, competing culture may have a negative effect on corporate environmental investment. A competing culture may lead to increased internal competition (Hitka et al., 2015) or even cut-throat competition and conflicts if managers cannot provide the right guidance (Andersen and Johansen, 2021). As a result, employee trust decreases and interpersonal relationships become tense, leading to splits within the company (Andersen and Johansen, 2021). Companies with such a working environment are highly associated with managers’ short-sightedness (Hu et al., 2021). According to upper echelons theory, managerial short-sightedness affects a firm’s investment decisions (Hu et al., 2021). Short-sighted managers are more inclined to invest in projects with short duration and high risks (Hu et al., 2021) than in long-term investment such as environmental investment (Wei and Zhou, 2020). Therefore, corporate competing culture may hinder environmental investment.

However, on the other hand, competing culture may also promote corporate environmental investment. First, as mentioned above, corporate competing culture increases internal competition, which, if properly guided by the managers, may increase employee motivation, productivity, and corporate market competitiveness (Fiordelisi et al., 2019). Corporate competitiveness is highly associated with firms’ social reputation (Barbosa and Oliveira, 2020; Bruna and Nicolò, 2020; Nguyen et al., 2021). Social reputation is one of the important social resources available to companies. Resource dependency theory suggests that to survive and gain an advantage over competition, companies shall rely on their external environment such as the social environment to get support (Pugliese et al., 2014). To a certain extent, the theory reveals the close relationship between firms and their external environment. Therefore, to improve social reputation and further acquire external resources and support, companies will actively practice social responsibility (Singh and Misra, 2020) and so invest more in pro-environmental activities, which enhances corporate image and increases their social value (Singh and Misra, 2020). Accordingly, a healthy competing culture may promote corporate environmental investment.

Second, to control speculative behaviors that may result from a competing culture (Hitka et al., 2015), managers will improve operation management and increase corporate compliance (Rocha and Salomão, 2019). Theory of compliance states that companies should comply with laws and regulations and establish sustainable development goals (Rocha and Salomão, 2019). Currently, companies are subject to increasingly stringent environmental laws and regulations, prompting them to increase environmental investment in order to improve resource use efficiency and reduce pollutant emissions (Tian et al., 2020). Firms increase environmental investment to make their production and operation more compliant and therefore lower the environmental compliance cost (Bierbaum et al., 2019). As such, corporate competing culture may promote environmental investment by increasing corporate compliance.

In summary, theory on managerial short-sightedness suggests that corporate competing culture gives rise to speculation that is detrimental to environmental investment; however, on the other hand, resource dependence theory and theory of compliance suggest that if a competing culture is well managed, firms will pay greater attention to social reputation and operational compliance, and therefore increase environmental investment. The mixed effects of corporate competing culture on environmental investment make the relationship between the two more valuable to investigate.

## Research Design

### Data

We use all Chinese A-share listed companies on the Shanghai and Shenzhen Stock Exchanges from 2010 to 2019 as the research setting. As financial firms follow different reporting schemes, we have eliminated financial firms from our sample. With missing data eliminated, we reach a final sample of 5, 915 firm-year observations.

The environmental investment data and control variables used in this study are derived from the *China Stock Market Accounting Research* (CSMAR); the management discussion and analysis (MD&A) disclosure data are derived from the MD&A database of the *Chinese Research Data Services Platform* (CNRDS); and the competing culture keywords are derived from the *WinGo Textual Analytics Database*.

### Measuring Corporate Competing Culture

Following Loughran and McDonald (2011), this paper adopts the keyword frequency method to measure corporate competing culture. There are two ways to determine word frequency—one is by the count of relevant words, and the other is by the percentage of the count of relevant words to the total word counts in the text (frequency ratio). To avoid the scale difference of absolute values (Loughran and McDonald, 2011), we follow Ferris et al. (2013) and Austin et al. (2021) and uses keyword frequency ratio to measure corporate competing culture. We construct the measure in following three steps.

*Step 1: Competing culture seed word selection.* Based on the dictionary constructed by Fiordelisi et al. (2019), we translated words with etyma related to the semantic meaning of “compete” *via* widely-used translation software into Chinese, and conducted a preliminary word screening on the translated Chinese words. Considering the different understanding of the semantic meaning of “compete” in Chinese and English, words unrelated to the semantic meaning of “compete” in Chinese were removed. For example, “agreem” in English has a connotation of “compete,” but it is translated as “agree, endorse, reach agreement” in Chinese. Therefore, the Chinese words translated from “agreem” were removed. In terms of etyma with multiple semantic meanings, for example, “mov” is translated as “motive” and “move” in Chinese, and “move” is associated with the semantic meaning of “compete” in Chinese, therefore, the translated Chinese word with the semantic meaning of “move” was retained.

We further supplemented the seed word set with synonyms using the Chinese Synonym Dictionary. For objectivity purpose, the revised seed word set was triangulated and examined by three experts in the field of corporate culture.

*Step 2: Near-synonym expansion.* In textual analysis, it is more effective to expand the seed word set with near-synonyms (Aghion et al., 2014). In this study, we used the deep learning technique provided by the *WinGo Textual Analytics Database* to expand the competing culture seed word set with near-synonyms. The deep learning technique uses the word2vec word embedding algorithm to convert cleaned texts into a set of vectors, and calculates the similarity between words. The greater the similarity, the smaller the difference of the semantic meaning between the two words. Therefore, we used the deep learning tool to derive an extended set of words with a similarity of 0.6 or more to the seed word set and removed the duplicate words^1^.

*Step 3: Calculating corporate competing culture.* Based on the extended seed word set, we calculated and used the competing culture seed word frequency ratio in the management discussion and analysis (MD&A) section of sample companies’ annual report to quantitively measure corporate competing culture. The greater the competing culture seed word frequency ratio, the stronger the competing culture of a company.

### Variables

This section introduces the dependent variable, explanatory variable, and control variables. Table 1 presents the descriptive statistics and the correlation coefficient matrix of all variables.

**TABLE 1:** Summary statistics deviations and correlation matrix.

Variable	Mean	SD	1	2	3	4	5	6	7	8	9	10	11
*1. Envir_inv*	15.21	2.29											
2*.Compete*	0.03	0.01	0.076										
3.*Roa*	1.48	0.78	0.022	–0.058									
4.*Size*	3.10	0.06	0.468	0.099	–0.121								
5.*Age*	2.76	0.38	0.069	–0.032	–0.074	0.193							
6.*TQ*	1.77	1.92	–0.269	–0.106	0.301	–0.541	–0.088						
7*.Envir_regu*	3.06	0.53	0.036	–0.095	0.084	0.069	0.163	–0.176					
8.*Independent*	38.30	10.16	–0.061	0.069	–0.036	–0.066	–0.130	0.100	–0.293				
9.*Ownership*	21.60	1.17	0.442	0.076	0.030	0.928	0.152	–0.439	0.095	–0.072			
10.*Leverage*	0.37	0.15	0.235	0.101	–0.402	0.488	0.150	–0.440	–0.040	–0.002	0.161		
11.*SOE*	0.52	0.50	0.140	0.203	–0.192	0.307	0.239	–0.176	–0.043	–0.018	0.216	0.321	
12.*Isduality*	0.22	0.41	–0.044	–0.105	0.058	–0.140	–0.058	0.095	0.027	0.026	–0.114	–0.113	–0.252

*Dependent variable.* The dependent variable is corporate environmental investment (*Envir_invest*). Following prior studies (e.g., Hu et al., 2017), we define the expenditure related to environmental protection in ongoing projects to measure corporate environmental investment.

*Explanatory variable.* The explanatory variable is corporate competing culture (*Compete*). We use the competing culture seed word frequency ratio in the MD&A texts of sample firms’ annual report as a measure of corporate competing culture.

*Control variables*. Following existing studies (e.g., Sloan, 1996; Shen et al., 2019; Su et al., 2020; Gu et al., 2021), we choose a set of firm characteristics as control variables, including firm age (*Age)*, firm size (*Size*), total liabilities (*Leverage*), return on assets (*Roa*), owner’s equity (*Ownership*), Tobin’s Q (*TQ*), state ownership (*SOE*), proportion of independent board members (*Inpendent*), and duality of the CEO (*Isduality)*. Moreover, because of the increasingly important influence of environmental regulation on companies’ green investment (Huang and Lei, 2021), the number of environmental regulations (*Envir_regu*) is also added as a control variable. Furthermore, we also control for industry fixed effects *(Industry FE)* and year fixed effects (*Year FE*).

## Results

### Corporate Competing Culture and Environmental Investment

To investigate the impact of competing culture on corporate environmental investment, we use the following regression model:


(1) $$E\,n\,v\,i\,r\_i\,n\,v\,e\,s\,t=\alpha_{0}+\alpha_{1}\,C\,o\,m\,p\,e\,t\,e+\beta \,Z+Y\,e\,a\,r\,F\,E+I\,n\,d\,u\,s\,t\,r\,y\,F\,E+\varepsilon$$

where *Envir_invest* represents corporate environmental investment, *Compete* is corporate competing culture, and *Z* denotes the control variables, including firm age (*Age*), firm size (*Size*), total liabilities (*Leverage*), return on assets (*Roa*), owners’ equity (*Ownership*), Tobin’s Q (*TQ*), percentage of independent board members (*Inpendent*), whether the CEO is also the chairman of the board (*Isduality*), whether the firm is state-owned (*SOE)*, and the number of environmental regulations (*Envir_regu*). In addition, we control for year fixed effects *(Year FE)* and industry fixed effects (*Industry FE*). The results are presented in Table 2.

**TABLE 2:** The impact of corporate competing culture on environmental investment.

Variables	Model 1	Model 2	Model 3	Model 4	Model 5	Model 6
*Compete*	25.5979***	10.9211***	15.4105***			18.4600**
	(3.6307)	(4.0123)	(4.6896)			(7.883)
*Compete1*				11.0520***		
				(3.0362)		
*Compete2*					15.0237**	
					(7.3872)	
*Roa*		0.2284***	0.2453***	0.2531***	0.2412***	0.2410***
		(0.0445)	(0.0513)	(0.0513)	(0.0515)	(0.0506)
*Size*		0.2323	0.4557*	0.4479*	0.4641**	0.3840**
		(0.2096)	(0.2331)	(0.2330)	(0.2335)	(0.1730)
*Age*		−0.2856***	−0.1960*	−0.2154*	−0.2013*	−0.2280*
		(0.1023)	(0.1166)	(0.1165)	(0.1167)	(0.1220)
*TQ*		−0.0356	−0.0307	−0.0272	−0.0344	−0.0337
		(0.0250)	(0.0294)	(0.0294)	(0.0294)	(0.0337)
*Envir_regu*		−0.1193	0.0689	0.0559	0.0725	−0.2190
		(0.1272)	(0.1465)	(0.1465)	(0.1467)	(0.1460)
*Qwnership*		0.5916***	0.4223**	0.4355**	0.4132**	0.3870**
		(0.1612)	(0.1788)	(0.1787)	(0.1792)	(0.1680)
*Independent*		−0.0052	−0.0075*	−0.0079**	−0.0075*	−0.00940**
		(0.0032)	(0.0039)	(0.0039)	(0.0039)	(0.0037)
*Leverage*		2.2481***	1.6413***	1.6851***	1.6564***	1.6600***
		(0.5559)	(0.6315)	(0.6311)	(0.6322)	(0.6020)
*SOE*		−0.0287	−0.1311	−0.1453*	−0.1200	−0.0819
		(0.0729)	(0.0853)	(0.0857)	(0.0856)	(0.0844)
*Isduality*		0.1285*	0.0131	0.0211	0.0061	0.1530*
		(0.0764)	(0.0905)	(0.0906)	(0.0906)	(0.0879)
*Constant*	14.4137***	3.0156	5.9578	5.5974	6.4372	−1.012
	(0.3964)	(3.7293)	(4.1509)	(4.1525)	(4.1545)	(1.1610)
*Year FE*	Yes	Yes	Yes	Yes	Yes	Yes
*Industry FE*	Yes	Yes	Yes	Yes	Yes	Yes
*No. of obs.*	5,912	4,035	2,722	2,722	2,722	2,770
*R* ^2^	0.1979	0.3529	0.3910	0.3916	0.3895	0.3910

(1) *, **, *** represent significant at the 10, 5, and 1% significance level, respectively; (2) standard deviations are provided in parentheses.

Model 1 controls for industry fixed effects and year fixed effects without adding any control variables. The results show that the impact of corporate competing culture on environmental investment is positive and statistically significant at the 1% significance level, indicating that corporate competing culture significantly promotes environmental investment. In Model 2, after controlling for firm age (*Age*), firm size (*Size*), total liabilities (*Leverage*), return on assets (*Roa*), owners’ equity (*Ownership*), Tobin’s Q (*TQ*), percentage of independent board members (*Inpendent*), duality of the CEO (*Isduality*), state ownership (*SOE)*, and the number of environmental regulations (*Envir_regu*), the coefficient of *Compete* is still significantly positive at the 1% significance level, which further confirms that competing culture has a significant and positive impact on corporate environmental investment. This is consistent with resource dependency theory that firms will gain more support from external environment by increasing environmental investment promoted by fostering a competing culture. The finding is also consistent with theory of compliance that a stronger competing culture will strengthen monitoring and regulation to circumvent speculative behavior and motivate companies to increase their environmental investment in order to reduce the cost of environmental regulation risks.

In addition, given the long-term nature of environmental investment, we follow Hu et al. (2017) and use the one-year-ahead corporate environmental investment as the dependent variable to re-estimate the model. The results, as shown in Model 3, illustrate that the impact of corporate competing culture on environmental investment is still significantly positive.

We further use two alternative measures of corporate competing culture—*Compete1* (the extended word set with a similarity of 0.5 or greater to the seed word set) and *Compete2* (the extended word set with a similarity of 0.7 or greater to the seed word set)—to re-estimate the regression models. The results, as shown in Models 4 and 5, are consistent with the baseline results. Therefore, our findings on the positive relationship between corporate competing culture and environmental investment are valid and robust.

Considering the reverse causality between corporate competing culture and environmental investment, we employ the system Generalized Moment Methods (Sys-GMM) to mitigate endogeneity concerns (Ferrell et al., 2016; Shi et al., 2016; Sutton et al., 2021). The results are displayed in Model 6 of Table 2. It results confirm the positive causal relationship between corporate competing culture and environmental investment. Besides, the results for underidentification test and weak instrument test both show the effectiveness of the instrument variable. Therefore, our key findings of the positive impact of competing culture on corporate environmental investment is reliable^2^.

### Heterogeneity Analysis

To explore the heterogeneous impact of size effect, region effect, governance effect, and industry effect, we examine the relationship between corporate competing culture and environmental investment in terms of different firm size (*scale*), geographical location (*east*), board size (*board*), and a binary variable of high-polluting industry (*pollu*). *scale* is taken as 1 if the firm size is smaller than the median value of firm size, and 0 otherwise. *east* is taken as 1 if the firm is located in the eastern China, and 0 otherwise. *board* is taken as 1 if the board size is larger than the median value of board size, and 0 otherwise. *pollu* is taken as 1 if the firm is in a heavy polluting industry, and 0 otherwise.

*Size effect.* As shown in the first two columns of Table 3, the coefficient of *Compete* is insignificant when a firm’s size is less than the median size of the sample firms (size < size_median), but significantly positive at the 5% significance level when the firm’s size is greater than the median size (size ≥ size_median). The results indicate that the positive impact of corporate competing culture on environmental investment is more pronounced in larger firms.

**TABLE 3:** Heterogeneity results.

Variables	*Scale*		*East*		*Board*		*Pollu*	
	SME	Large	Central & Western	Eastern	Small	Large	Low	High
	(1)	(2)	(3)	(4)	(5)	(6)	(7)	(8)
*Compete*	8.7425	11.3265**	25.7425***	−0.6012	8.7607	15.5482**	2.5915	16.6036***
	(5.7640)	(5.6673)	(5.6511)	(5.6401)	(5.4379)	(6.0806)	(6.2773)	(5.2226)
*Roa*	0.1394**	0.2872***	0.1401**	0.2833***	0.2733***	0.1639**	0.3110***	0.1961***
	(0.0620)	(0.0676)	(0.0620)	(0.0642)	(0.0610)	(0.0669)	(0.0739)	(0.0557)
*Size*	19.3683***	−7.2482	−0.7252	8.6798	2.7366	7.6158	−8.4408	10.6864**
	(5.2267)	(5.9661)	(4.8622)	(5.8206)	(5.8091)	(5.1448)	(6.5606)	(4.5801)
*Age*	−0.4109***	−0.0552	0.0824	−0.4952***	−0.3109**	−0.2596	−0.3541**	−0.2256*
	(0.1287)	(0.1709)	(0.1584)	(0.1403)	(0.1300)	(0.1716)	(0.1584)	(0.1345)
*TQ*	−0.0670**	0.0016	−0.0026	−0.0605	−0.0428	−0.0251	−0.0049	−0.0519*
	(0.0298)	(0.0681)	(0.0331)	(0.0377)	(0.0330)	(0.0398)	(0.0418)	(0.0313)
*Envir_regu*	−0.2585	0.0280	−0.5340**	−0.1277	−0.1565	0.0129	−0.1629	−0.0941
	(0.1729)	(0.1850)	(0.2442)	(0.1655)	(0.1681)	(0.1974)	(0.1995)	(0.1644)
*Ownership*	−0.0328	1.1187***	0.8328***	0.3241	0.5939**	0.5144**	1.1243***	0.3185
	(0.2193)	(0.2488)	(0.2081)	(0.2529)	(0.2556)	(0.2150)	(0.2886)	(0.1944)
*Independent*	0.0010	−0.0137***	0.0006	−0.0110**	−0.0032	−−0.0084	0.0011	−0.0095**
	(0.0042)	(0.0048)	(0.0047)	(0.0043)	(0.0041)	(0.0056)	(0.0048)	(0.0042)
*leverage*	0.3957	4.1887***	3.1841***	1.5898*	2.5488***	1.4339*	4.3409***	0.9933
	(0.7000)	(0.9447)	(0.7687)	(0.8138)	(0.7985)	(0.8332)	(0.9651)	(0.6802)
*SOE*	−0.0029	−0.0353	0.0933	0.0486	0.0640	−0.1672	−0.1040	0.0140
	(0.1010)	(0.1064)	(0.1094)	(0.1052)	(0.0971)	(0.1142)	(0.1203)	(0.0915)
*Isduality*	0.1368	0.0841	0.2444**	0.0385	0.0811	0.1761	−0.1783	0.3407***
	(0.0959)	(0.1261)	(0.1207)	(0.0994)	(0.0979)	(0.1261)	(0.1254)	(0.0967)
*Constant*	−44.0780***	14.0946	-0.6425	−19.3291	−5.9307	−18.2564	17.3302	−24.6280**
	(11.7187)	(13.0829)	(10.5398)	(12.5498)	(12.4046)	(11.3041)	(14.0164)	(9.9526)
*Year FE*	Yes	Yes	Yes	Yes	Yes	Yes	Yes	Yes
*Industry FE*	Yes	Yes	Yes	Yes	Yes	Yes	Yes	Yes
*No. of obs.*	1,900	2,135	1,787	2,248	2,315	1,720	1,606	2,429
*R−squared*	0.2810	0.3169	0.4260	0.3524	0.3363	0.3956	0.3896	0.3230

(1) *, **, *** represent significant at the 10, 5, and 1% significance level, respectively; (2) standard deviations are provided in parentheses.

*Region effect.* As shown in Columns 3 and 4 of Table 3, the coefficient of *Compete* is significantly positive at the 1% significance level in firms in central and western China, but not significant in firms in eastern China. The finding indicates that in less developed regions, firms with a competing culture are more likely to invest in environmental protection.

*Governance effect.* As shown in Columns 5 and 6 of Table 3, the coefficient of *Compete* is insignificant when the board size is less than the median board size, but significantly positive at the 5% significance level when the board size is greater than the median value. The results illustrate that the stronger the corporate governance, the more pronounced the positive impact of competing culture on its environmental investment.

*Industry effect.* As shown in the last two columns of Table 3, the coefficient of *Compete* is significantly positive at the 1% significance level in high-polluting firms, but not significant in low-polluting firms. Corporate investment decisions, including investment in environmental protection and pollution control, are affected by environmental laws and regulations (Gray and Deily, 1996). When a firm has a strong competing culture, managers are more likely to strengthen corporate governance and compliance to avoid the speculative conduct that the competing culture may cause (Hitka et al., 2015). This is more common in heavy polluting companies as heavy polluters face stricter restrictions on environmental laws and regulations and so higher penalty costs. Therefore, the impact of corporate competing culture on environmental investment is more pronounced in heavy polluting companies.

### Mediating Analysis

Management team with short-sightedness may reduce environmental investment for personal gains as environmental investment cannot guarantee a short-term payoff (Wei and Zhou, 2020). However, high-quality internal control might effectively reduce short-sighted decisions (Cheng et al., 2013) and promote better social responsibility (Bierbaum et al., 2019), thereby increasing the scale of corporate environmental investment. Meanwhile, corporate culture is closely related to the quality of internal control (Yu et al., 2021). Therefore, the quality of a firm’s internal control might transmit the positive impact of corporate competing culture onto its environmental investment.

To explore the channel in the relationship between corporate competing culture and environmental investment, we use the internal control index obtained from the internal control database created by the *DIB Database* as a measure of internal control quality to examine its mediating effect in the competing culture-environmental investment nexus.

As displayed in Table 4, the coefficient of *Compete* in the first column is positive and statistically significant, and the coefficient of *ici* (internal control index) in the second column is also positive and significant, indicating that corporate competing culture promotes environmental investment through the enhancement of internal control quality. Therefore, firms’ internal control quality transmits the positive impact of corporate competing culture onto environmental investment.

**TABLE 4:** Mediating analysis of internal control quality.

*Variables*	*ici*	*envir_invest*
*Compete*	1,039.4768***	10.9472***
	(282.2982)	(4.0952)
*ici*		0.0004*
		(0.0002)
*Roa*	30.2879***	0.2136***
	(3.1021)	(0.0455)
*Size*	−101.4917***	0.2852
	(14.6230)	(0.2131)
*Age*	1.8930	−0.2686**
	(7.4645)	(0.1081)
*TQ*	−4.6751***	−0.0319
	(1.7733)	(0.0257)
*Envir_regu*	−0.5086	−0.1300
	(8.9694)	(0.1299)
*Ownership*	107.8710***	0.5410***
	(11.2367)	(0.1647)
*Independent*	−0.0485	−0.0059*
	(0.2243)	(0.0032)
*Leverage*	245.5929***	2.1172***
	(39.1321)	(0.5696)
*SOE*	−16.1487***	−0.0223
	(5.1194)	(0.0743)
*Isduality*	−14.4266***	0.1339*
	(5.4186)	(0.0786)
*Constant*	−1,766.5930***	3.9113
	(260.3035)	(3.7928)
*Year FE*	Yes	Yes
*Industry FE*	Yes	Yes
*No. of obs.*	3,914	3,912
*R* ^2^	0.1847	0.3511

(1) *, **, *** represent significant at the 10, 5, and 1% significance level, respectively; (2) standard deviations are provided in parentheses.

## Conclusion

Using Chinese listed firms from 2010 to 2019 as the research setting, this paper develops a quantitative measure of corporate competing culture through textual analysis and examines the impact of corporate competing culture on environmental investment. The results show that (1) corporate competing culture has a significant and positive impact on environmental investment; (2) the results remain robust to alternative measures of corporate competing culture and environmental investment; (3) the positive impact of corporate competing culture on environmental investment is more pronounced in companies with larger size, stronger corporate governance, in high-polluting industry, and located in less developed regions; (4) internal control quality plays a mediating role in transmitting the impact of corporate competing culture onto environmental investment.

This study has important practical implications. First, it broadens the research in the area of corporate sustainability by providing empirical evidence that corporate competing culture contributes to corporate sustainability by promoting environmental investment. Second, internal control quality serves as an important channel for the positive impact of corporate competing culture on environmental investment; firms can thus promote the positive effect of competing culture on environmental investment by improving the quality of internal control. Third, we use deep learning technique to measure corporate competing culture and contribute to the quantitative measurement of corporate competing culture.

## Data Availability Statement

The original contributions presented in the study are included in the article/supplementary material, further inquiries can be directed to the corresponding author.

## Author Contributions

JT, WC, QC, YH, and SH contributed to conception and design of the study. JT and WC organized the database and performed the statistical analysis. All authors wrote the first draft of the manuscript, contributed to manuscript revision, read, and approved the submitted version.

## Conflict of Interest

The authors declare that the research was conducted in the absence of any commercial or financial relationships that could be construed as a potential conflict of interest.

## Publisher’s Note

All claims expressed in this article are solely those of the authors and do not necessarily represent those of their affiliated organizations, or those of the publisher, the editors and the reviewers. Any product that may be evaluated in this article, or claim that may be made by its manufacturer, is not guaranteed or endorsed by the publisher.

## Footnotes

1 To test the robustness of our results, we built three competing culture word sets: (1) the extended word set (Compete1) with a similarity of 0.5 or greater to the seed word set, (2) the extended word set (Compete) with a similarity of 0.6 or greater to the seed word set, and (3) the extended word set (Compete2) with a similarity of 0.7 or greater to the seed word set. We use Compete1 in the baseline regressions, and conducted robustness checks using the remaining two word sets as alternative measures of corporate competing culture.

2 The results are available upon request.
